# SS_CASE_UNet: an attention-enhanced semi-supervised framework for fetal cerebellum segmentation in ultrasound images

**DOI:** 10.1038/s41598-025-28201-4

**Published:** 2025-12-24

**Authors:** Amene Vatanparast, Mansoor Fateh, Hoda Mashayekhi, Saideh Ferdowsi

**Affiliations:** 1 Faculty of Computer Engineering, Shahrood University of Technology, Shahrood, Iran; 2 School of Mathematics, Statistics and Actuarial Science (SMSAS), University of Essex, Colchester, UK

**Keywords:** Fetal cerebellar segmentation, Ultrasound image, Semi-supervised learning, Convolution neural network, Attention block, Squeeze-and-excitation block, Computational biology and bioinformatics, Engineering, Health care, Mathematics and computing, Medical research

## Abstract

Accurate segmentation of the fetal cerebellum in ultrasound images is crucial for assessing fetal development and detecting prenatal abnormalities. However, this task remains challenging due to factors such as image noise, complex anatomical structures, and limited availability of annotated data, which is further compounded by the high cost and effort required for manual labeling. To address these challenges, we propose SS_CASE_UNet, a novel semi-supervised segmentation framework that enhances U-Net with attention mechanisms to better manage image noise and anatomical complexity. Additionally, a multi-stage semi-supervised training strategy effectively mitigates the scarcity of annotated data. The architecture integrates Squeeze-and-Excitation blocks for dynamic channel-wise feature recalibration and a Coordinate Attention block at the bottleneck to capture precise spatial and long-range dependencies. Our multi-stage training pipeline leverages both labeled and unlabeled data through iterative pseudo-label and re-training, improving generalization in low-annotation scenarios. Experimental results demonstrate that SS_CASE_UNet outperforms existing methods, achieving a Dice Similarity Coefficient (DSC) of 87.65%, along with high accuracy (99.08%), precision (93.49%), recall (82.34%), and Jaccard Similarity (81.78%). Despite incorporating advanced attention mechanisms, our model maintains a balanced complexity-performance trade-off. These results highlight SS_CASE_UNet as a robust and clinically practical solution for automated segmentation of the fetal cerebellum in ultrasound images.

## Introduction

Ultrasound (US) imaging is a cornerstone of prenatal diagnostics, offering a non-invasive, real-time, and cost-effective approach for assessing fetal development. Unlike other imaging techniques, such as Computed Tomography (CT) or Magnetic Resonance Imaging (MRI), ultrasound does not expose the fetus to ionizing radiation, making it a safer option for routine examinations throughout pregnancy^1^. Its accessibility and portability further contribute to its widespread adoption, particularly in resource-limited settings, enabling early detection of potential anomalies and guiding clinical interventions^2^. Additionally, the dynamic nature of ultrasound facilitates the visualization of organ movement and blood flow, offering essential functional information in conjunction with anatomical details.

**Fig. 1:**
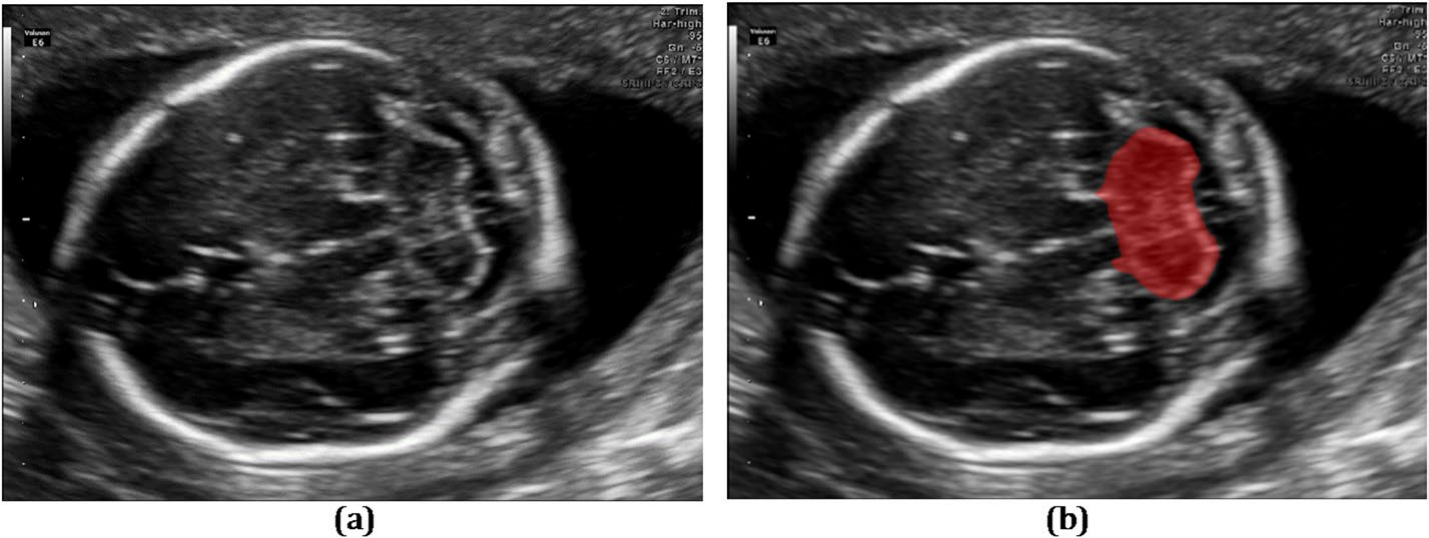
The cerebellar region in an ultrasound image. (a) Original image. (b) Highlighted cerebellar area.

Accurate evaluation of the fetal brain is essential for identifying neurological abnormalities and ensuring normal developmental progression. Standard prenatal ultrasound protocols typically involve acquiring and classifying specific fetal brain planes, including trans-cerebellar, trans-ventricular, and trans-thalamic views^3,4^. However, variability in fetal position, maternal body habitus, and image quality can complicate this process, highlighting the need for advanced image analysis techniques^5–7^.

Among various fetal brain structures, the cerebellum plays a significant role in development, contributing to motor control, coordination, and early cognitive functions^8^. Its proper formation indicates neurological health, while abnormalities may signal developmental disorders. However, accurately segmenting the fetal cerebellum in ultrasound images remains particularly challenging. This difficulty arises from its small size, complex anatomical folding, rapid morphological changes across gestational ages, and the presence of surrounding hyperechoic tissues^9^. Additional challenges include blurred or ambiguous ultrasound boundaries, noise-related data loss, and visual similarity to adjacent structures, such as the amniotic fluid and uterine wall^10^. Figure 1 illustrates the cerebellar region in a trans-cerebellar ultrasound image.

Manual segmentation, although widely used, is labor-intensive, time-consuming, and requires significant sonographic expertise^11,12^. These limitations highlight the urgent need for robust and automated cerebellum segmentation methods to ensure accurate, efficient, and reproducible prenatal assessments.

In the medical imaging field, Convolutional Neural Networks (CNNs) have demonstrated remarkable effectiveness, leading to significant advancements in the detection and segmentation of various anatomical structures and pathologies^13–15^. Notable successes include applications in musculoskeletal ultrasound^16^ and fetal imaging^5^, underscoring the transformative potential of deep learning in automating and enhancing clinical diagnostic workflows^17^.

Despite these successes, directly applying neural network architectures designed for natural image datasets to the medical imaging field presents considerable challenges. Medical image datasets differ in several key aspects: their applications are narrowly specialized, data acquisition requires stringent ethical approvals, and accurate labeling demands extensive expertise from clinicians, making the process time-consuming and expensive^18^. Consequently, medical research datasets are often small, resulting in data scarcity that presents a significant challenge for deep convolutional neural network (DCNN) models. While DCNNs provide clear advantages for medical image analysis, their application in ultrasound image segmentation remains relatively underexplored. This gap highlights the need for further research focused on optimizing DCNNs for the unique characteristics of ultrasound-based segmentation.

Accurate segmentation of the fetal cerebellum in ultrasound images is essential for assessing fetal development, estimating gestational age, and detecting central nervous system abnormalities. However, manual segmentation is time-consuming and prone to inter- and intra-observer variability, highlighting the need for automated approaches. As a result, fetal cerebellum segmentation has become a central focus of research. Early studies utilized traditional image processing and statistical shape models, while recent advancements have increasingly embraced deep learning, particularly convolutional neural networks, to achieve higher accuracy and greater automation.

Fetal cerebellum segmentation methodologies can be broadly categorized into two main streams: traditional model-based approaches and data-driven deep learning techniques.

### Traditional model-based approaches

Initial efforts in automated fetal cerebellum segmentation often utilized statistical shape models. These methods typically involve constructing a deformable model of the cerebellum from a training dataset, which is then fitted to new ultrasound images.

One such approach is the use of 3D statistical shape models, which represent the cerebellum’s 3D shape through parameters that capture typical variations. For instance, Fanti et al. proposed a 3D segmentation system utilizing a Point Distribution Model (PDM) based on spherical harmonics (SPHARMs)^19^. This PDM was automatically adjusted through an objective function optimized by gray-level voxel profiles, often employing a genetic algorithm. To assist with initialization and plane selection, they integrated a CNN (YOLO v2) for cerebellum detection on each plane. They reported a Dice coefficient of 0.83 ± 0.10 and a Hausdorff distance of 3.61 ± 0.83 mm across 18 ultrasound volumes.

The primary advantage of statistical shape models is their ability to encode prior anatomical knowledge, resulting in robust segmentations even under challenging conditions. However, a significant drawback is their reliance on accurately constructed shape models, which may struggle with atypical anatomies or poor image quality that deviates from the training data. Furthermore, inherent challenges in 3D fetal ultrasound, such as acoustic shadow artifacts, speckle noise, and intensity inhomogeneity from fetal movement, pose additional limitations. Similarly, Velásquez et al.^20^ explored automatic cerebellum segmentation using a spherical harmonics model, achieving a mean Dice coefficient of 0.689 on 10 ultrasound volumes. While their approach is structured, performance can be sensitive to initialization and dataset variability, with artifacts limiting precision without robust pre-processing. Beyond statistical modeling, earlier pattern recognition methods that utilized cerebellar model articulation controllers with fractal features^21^ demonstrated strong domain-specific performance. However, they often struggled to generalize to the variability and noise present in medical ultrasound images.

### Deep learning-based approaches

The advent of deep learning has revolutionized medical image segmentation, providing powerful data-driven solutions that learn complex features directly from raw image data^22^.

Many recent studies have adopted Convolutional Neural Networks, particularly enhanced variants of the U-Net architecture, due to their effectiveness in medical image segmentation tasks. The U-Net architecture features an encoder-decoder structure with skip connections, making it well-suited for capturing both high-level semantic information and fine-grained spatial details, which are crucial for accurate segmentation.

Shu et al.^23^ introduced FCRB U-Net (Fully Connected Residual Block U-Net), an enhanced version of the U-Net that replaces standard double convolution operations with fully connected residual blocks. They also incorporated an effective channel attention module to improve feature extraction and utilized a feature reuse module during the decoding stage. This design aims to reduce feature information loss during convolution and enhance segmentation accuracy. They reported an average IoU of 86.72% and a mean Dice index of 90.45%, demonstrating significant improvements over basic U-Net models. A noted disadvantage is the challenge of sample imbalance and the necessity for large-scale, high-quality annotated datasets for fully supervised training.

Wang et al.^18^ presented ECAU-Net (Efficient Channel Attention U-Net) for fetal ultrasound cerebellum segmentation. Their method builds on the U-Net backbone and integrates Efficient Channel Attention (ECA) modules, which utilize one-dimensional convolutional layers with shared parameters to significantly reduce the number of model parameters without sacrificing performance. This enhances the model’s efficiency for deployment on resource-constrained platforms. They developed their own JSUAH-Cerebellum US database and performed data augmentation. ECAU-Net achieved a mean Jaccard Similarity (JS) of 86.01% and a Dice Similarity Coefficient (DSC) of 91.35%.

Chen et al.^9^ proposed ResU-Net-c, a semantic segmentation model specifically optimized for 2D fetal ultrasound brain images of the cerebellum. Their method builds on the U-Net architecture by integrating residual blocks and introducing dilated convolutions in the later layers to enhance feature extraction and preserve spatial details. Trained and evaluated on a dataset of 588 images for training and 146 for testing (using 5-fold cross-validation), ResU-Net-c achieved a mean Dice Score Coefficient (DSC) of 87.00% and a Hausdorff Distance (HD) of 28.15. The authors acknowledge that the inherent characteristics of ultrasound images, such as noise and varying image quality, continue to pose significant challenges to accurate segmentation. Table 1 presents a comparative analysis of deep learning methodologies for fetal cerebellar segmentation.

**Table 1:** Comparative Article in fetal cerebellar segmentation (deep learning methods).

Article	Method	Number of Images	Metrics
^18^ (2022)	ECAU-Net, U-Net variant with efficient channel attention	JSUAH-Cerebellum US database (private)	Mean Jaccard Score (JS): 86.01%, Dice Similarity Coefficient (DSC): 91.35%
^23^ (2022)	FCRBU-Net, U-Net variant with fully connected residual blocks and channel attention	JSUAH-Cerebellum US database (private)	Average IoU: 86.72%, Mean Dice Index: 90.45%
^11^ (2025)	Differential CNN for localization, Dual CNN for segmentation	Not explicitly stated for the cerebellum	Accuracy (plane localization): 98.6%, Dice Coefficient (DSC) (cerebellum segmentation): 0.956

#### Multi-task learning and dual

Some researchers have explored multi-task learning, where a single network is trained to perform multiple related tasks simultaneously, as well as dual-network approaches to enhance overall performance.

Namburete et al.^24^ investigated a Multi-task CNN for Structural Semantic Segmentation in 3D Fetal Brain Ultrasound. This approach aimed to segment multiple fetal brain structures, including the white matter, thalamus, brainstem, and cerebellum, from 3D ultrasound images using atlas-generated labels. This method allows the network to learn shared representations across different structures, potentially improving the accuracy of individual segmentations. Trained on 480 volumes and tested on 48, their multi-task CNN achieved a Dice coefficient of over 0.77 for cerebellum segmentation and 0.93 for white matter. A potential disadvantage, though not explicitly stated, could be the increased complexity of annotation for multiple structures and the challenge of ensuring uniform performance across all segmented regions, especially if some structures are harder to define or appear less frequently.

Vetriselvi et al.^11^ introduced a dual approach to plane localization and cerebellum segmentation in ultrasound images. This work addresses two critical steps: accurate plane localization (finding the correct anatomical view) and subsequent segmentation. They proposed two specialized CNN architectures: a “differential CNN” for plane localization, incorporating diverse convolutional operators, and a “dual CNN” for cerebellum segmentation, which integrates both original image data and complementary feature maps. This dual strategy aims to improve the overall diagnostic pipeline. The proposed models demonstrated high performance, achieving 98.6% accuracy for plane localization and a Dice coefficient of 0.956 for cerebellum segmentation, although specific dataset sizes were not detailed in the snippets. While effective, the complexity of a dual-network system could pose challenges for real-time applications or computational resources.

#### Biometric measurement-focused studies

It is essential to differentiate between papers focused on segmentation and those concentrating on biometric measurements using ultrasound^25^. Manzo et al.^26^ aimed to establish reference ranges for the fetal transverse cerebellar diameter and cerebellar area for gestational age estimation. While this work is crucial for clinical practice, it does not present an automated segmentation method; instead, it relies on manual or semi-manual measurements to derive biometric data. The limitation lies not in the technique itself but in the dependence on labor-intensive measurement processes that automated segmentation seeks to address. Their study on 384 pregnancies demonstrated strong correlations (e.g., *r* = 0.89 for the cerebellar area with gestational age), validating transverse cerebellar diameter as a reliable estimator of gestational age. Still, it does not directly contribute to the methodologies of automatic segmentation.

Wang et al.^27^ presented a framework for the automatic localization and quantitative segmentation of the cavum septum pellucidum complex (CCC) and the cerebellar vermis (CV) in fetal brain ultrasound images. Their approach employs a variational autoencoder to generate average templates, followed by a multi-step localization and segmentation strategy based on morphological characteristics. This framework, which leverages deep learning for landmark localization, aims to automate fetal brain biometry. Validated on 140 fetal brain mid-sagittal ultrasound images, it demonstrated good localization accuracy, with automated measurements typically differing from manual ones by within 1–3 mm. A key limitation noted is the inherent challenge of segmenting complex structures, such as the CCC and CV, along with issues including fetal motion and the lack of consensus on biometric definitions.

Despite significant advancements in automated fetal ultrasound image analysis, several challenges persist. Deep learning methods generally require large, high-quality annotated datasets, which are difficult and costly to obtain in medical imaging. This leads to issues such as sample imbalance and limited generalization to unseen data. While many U-Net variants have been proposed, few effectively address the subtle, noise-sensitive characteristics of fetal brain ultrasound images through architectural refinement and semi-supervised learning. Consequently, existing models often struggle with precise boundary delineation, especially for small or low-contrast structures like the fetal cerebellum.

Our work introduces the SS_CASE_UNet, a novel semi-supervised segmentation method based on an attention-enhanced U-Net architecture. This approach is designed to directly address the most critical challenges in fetal cerebellum ultrasound (US) image segmentation, including image noise, the complexity of anatomical folding, and ambiguous boundaries. The model is specifically engineered to manage the cerebellum’s small size and dynamic morphological changes by effectively capturing fine-grained spatial and contextual features. Importantly, the SS_CASE_UNet overcomes the established weaknesses of manual segmentation, which are time-consuming, labor-intensive, and prone to significant inter- and intra-observer variability. By intelligently leveraging a large pool of unlabeled data alongside limited labeled examples, our method aims to achieve superior segmentation performance and generalization across diverse images.

It’s important to acknowledge that leveraging unlabeled data is central to other advanced methods, such as the ASC (Appearance and Structure Consistency) framework^28^. However, our core objectives and technical applications differ significantly. ASC focuses on Unsupervised Domain Adaptation (UDA) for fetal brain MRI segmentation, where the primary challenge is bridging domain shifts between different datasets. In contrast, our SS_CASE_UNet is a Semi-Supervised Learning solution specifically designed for fetal cerebellum segmentation in noisy ultrasound images. Our focus is on mitigating data scarcity by creating and rigorously filtering high-confidence pseudo-labels from the unlabeled ultrasound data.

Another relevant approach that utilizes unlabeled data involves general-purpose SSL frameworks, such as the Semi-Supervised Learning with Pseudo-Label Correction (SEMI-PLC)^29^. This method addresses data scarcity through a dual-subnet architecture trained with Consistency Loss (PLC). The key distinction lies in the pseudo-label generation and filtering mechanism: SEMI-PLC forms a consensus prediction from its two subnets and filters the resulting pseudo-label using both a stability mask (which checks robustness to perturbations) and a confidence mask. In contrast, our approach employs a distinct and refined strategy for iterative pseudo-label generation, specifically designed to better cope with the unique and highly variable noise characteristics present in fetal ultrasound imaging.

In this paper, we present the SS_CASE_UNet methodology, designed to extract highly discriminative features from noisy ultrasound images while intelligently expanding the effective training dataset by leveraging unlabeled data. This strategy aims to significantly enhance segmentation accuracy, improve model generalization, and provide a more reliable tool for clinical assessment and diagnosis. The main contributions of this work are summarized as follows:

- Attention-Enhanced U-Net Architecture (CASE_UNet): We introduce a U-Net architecture specifically tailored for fetal cerebellum segmentation. This design incorporates Squeeze-and-Excitation (SE) blocks in the encoder and decoder paths, as well as a Coordinate Attention (CA) block at the bottleneck. This dual attention mechanism allows the model to dynamically recalibrate channel-wise features (SE) and capture precise positional and long-range dependencies (CA), resulting in a more discriminative feature representation that is crucial for challenging ultrasound data.
- Robust Semi-Supervised Training Pipeline: We propose and implement a multi-stage semi-supervised learning strategy that effectively leverages unlabeled ultrasound images. Through a rigorous pseudo-labeling and iterative re-training process, our pipeline significantly enhances model robustness and generalization, directly addressing the scarcity of expert-annotated medical data.
- Context-Preserving Data Augmentation: To further mitigate data limitations and improve model invariance to real-world variations, we employ a comprehensive data augmentation strategy. This includes noise reduction techniques, morphological operations on masks to refine structural details, and diverse geometric transformations (e.g., zoom, flips), all of which substantially expand the effective training dataset while preserving essential anatomical context.
- Optimized and Rigorous Evaluation Framework: Our framework features an optimized hybrid Binary Cross-Entropy and Dice loss function for robust training. The multi-stage training regimen encompasses decoder training, full network fine-tuning, and subsequent pseudo-label-driven refinement. Performance is rigorously evaluated using standard metrics (Accuracy, Dice, Jaccard, etc.), complemented by Test-Time Augmentation (TTA) to ensure highly reliable and robust segmentation outcomes.

The remainder of this paper is organized as follows: “Proposed method” provides a detailed description of the SS_CASE_UNet methodology. “Result analysis” presents the experimental setup, evaluation methods, and results. “Discussions” includes a thorough discussion of the findings. Finally, “Conclusion” offers the conclusion.

## Proposed method

Our proposed methodology for fetal cerebellum segmentation from prenatal ultrasound images is based on a novel multi-stage semi-supervised learning strategy. This approach directly addresses the fundamental challenge of limited labeled medical data by effectively combining a robust attention-enhanced U-Net architecture with a structured pipeline for utilizing unannotated images. The central innovation of the SS_CASE_UNet is its ability to maximize the utility of all available data, achieving superior segmentation accuracy and model generalization in this challenging clinical context.

### Model architecture: CASE_UNet

The foundational architecture of our segmentation framework is the U-Net, a well-established encoder-decoder Convolutional Neural Network known for its effectiveness in biomedical image segmentation, especially in scenarios with limited annotated data^30^. The design of the U-Net facilitates precise pixel-wise localization by utilizing skip connections to merge rich contextual information from the contracting (encoder) path with high-resolution spatial details from the expansive (decoder) path.

**Fig. 2:**
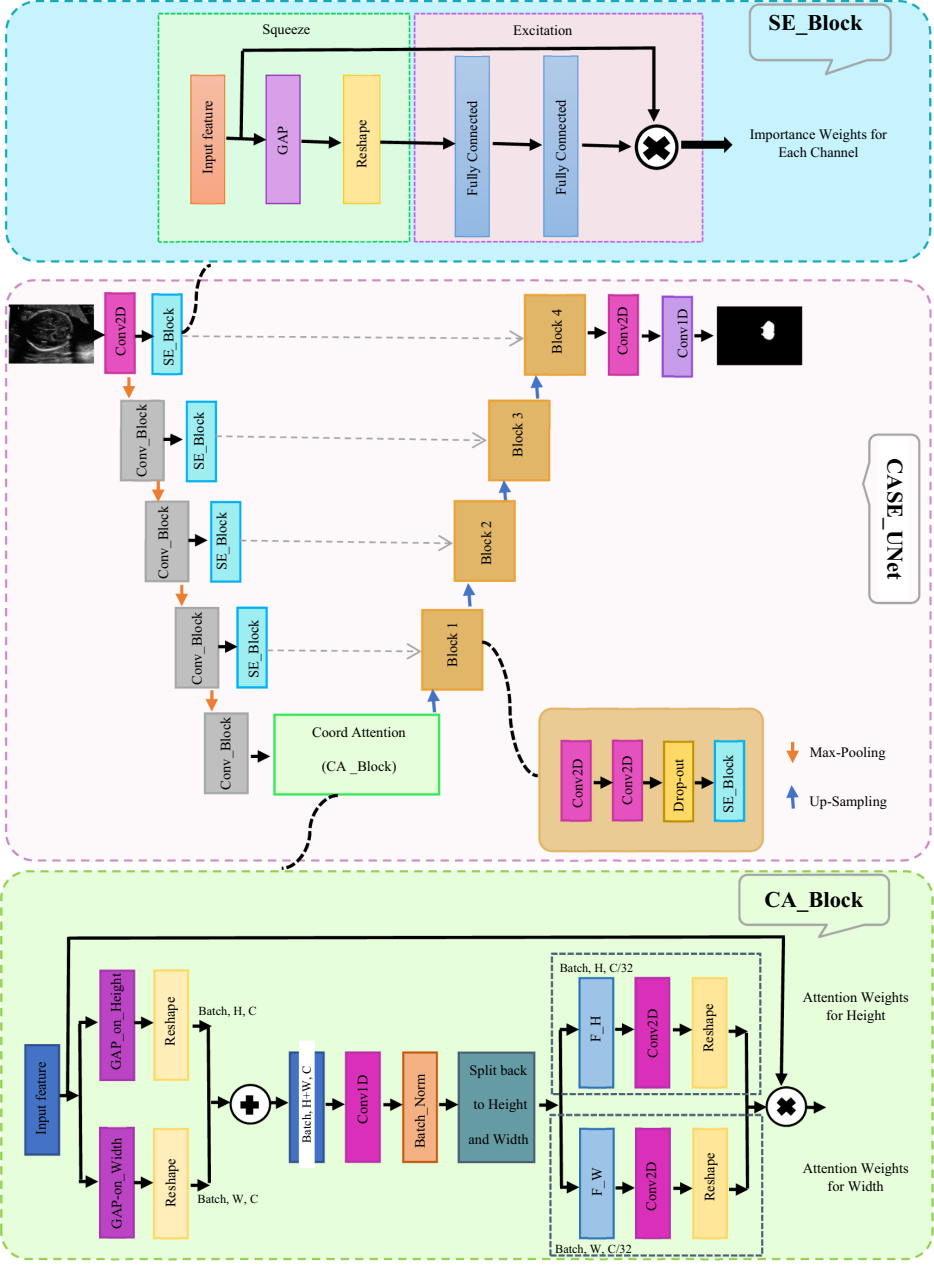
The schema of the CASE_UNet model.

To significantly enhance the U-Net’s ability to differentiate between complex anatomical structures and the inherent noise of ultrasound images, we introduce CASE_UNet. This novel architecture incorporates Squeeze-and-Excitation (SE) blocks in both the encoder and decoder paths, along with a Coordinate Attention (CA) block at its bottleneck (as illustrated in Fig. 2). This integration allows for dynamic channel-wise feature recalibration via the SE blocks and enables the capture of precise positional and inter-channel dependencies through the CA block, resulting in a more discriminative and contextually aware feature representation.

Encoder path (contracting path): The encoder extracts multi-scale, hierarchical feature representations and captures comprehensive contextual information from input ultrasound images. In our implementation, we utilize a pre-trained EfficientNetB0 model as the backbone. EfficientNetB0 is known for its balanced scaling of depth, width, and resolution, making it an efficient and accurate feature extractor. Using an ImageNet pre-trained backbone significantly enhances transfer learning, accelerating convergence and improving the model’s ability to learn robust, generalizable features from diverse natural image datasets, which can then be effectively adapted to our specific medical imaging domain.

To enhance feature recalibration and enrich the information transferred to the decoder, an SE block is integrated after the output of each major convolutional block, immediately preceding the skip connection. This strategic placement ensures that the feature maps sent to the expansive path are highly discriminative.

The Squeeze-and-Excitation (SE) block^31^ enhances neural network features by dynamically recalibrating them, allowing the model to emphasize the most relevant feature maps for a given task, such as segmentation. The process begins with the “squeezing” phase, in which each channel’s spatial information is condensed into a descriptor using global average pooling. For an input feature map$\:\:X\: \in \:{R}^{H\times\:W\times\:C}\:$, the squeeze operation $\:{F}_{sq}\left(X\right)$ generates a channel descriptor $\:z\: \in \:{R}^{1\times\:1\times\:C},\:$where each element $\:{z}_{c}$ is computed in Eq. (1).

1 $$\:{z}_{c}=\:{F}_{sq}\left({X}_{c}\right)=\frac{1}{H\times\:W}\:{\sum\:}_{i=1}^{H}{\sum\:}_{j=1}^{W}{X}_{c}\:(i,j)$$

Next, during the “excitation” phase, two fully connected layers learn the importance weights for each channel. These learned weights are then applied to scale the original feature map, effectively highlighting crucial features while diminishing the impact of less significant ones. The excitation operation $\:{F}_{ex}\left(z\right)\:$Calculates the channel-wise attention weights $\:s\: \in \:{R}^{C}$ in Eq. (2).

2 $$\:s=\:\:{F}_{ex}\left(z,\:{W}_{1},{W}_{2}\:\right)=\sigma\:\left({W}_{2}\delta\:\right({W}_{1}z\left)\right)$$

Finally, the recalibrated output feature map $\:{X}_{out}$ is obtained by element-wise multiplication of the original input feature map X with the learned attention weights s, as shown in Eq. (3).

3 $$\:{X}_{out}=\:\:X\:\otimes\:s$$

This dynamic re-weighting improves model accuracy with minimal computational overhead, making the SE block an effective addition to many convolutional neural networks.

Bottleneck: The deepest layer of the encoder acts as the bottleneck, providing the most semantically rich and spatially compressed feature representation. At this crucial stage, we introduce a Coordinate Attention (CA) block^32^, designed to enhance neural networks by accurately identifying important features along both height and width dimensions. Unlike traditional global pooling, the CA block factorizes the process into two separate one-dimensional feature encoding processes for horizontal and vertical coordinates. This mechanism enables the model to effectively capture long-range spatial dependencies while simultaneously preserving precise positional information—an essential capability for complex boundary differentiation in ultrasound images. For an input feature map $\:X\: \in \:{R}^{H\times\:W\times\:C}$, the process involves.

1. 1D Global Pooling:

- Horizontal pooling in Eq. (4).

4 $$\:{z}_{h}^{c}\left(h\right)=\:\frac{1}{W}\:{\sum\:}_{j=1}^{W}{x}_{c}(h,j)\:(\mathrm{y}\mathrm{i}\mathrm{e}\mathrm{l}\mathrm{d}\mathrm{i}\mathrm{n}\mathrm{g}\:{Z}_{h}\: \in \:{R}^{H\times\:1\times\:C}$$

- Vertical pooling in Eq. (5).

5 $$\:{z}_{w}^{c}\left(w\right)=\:\frac{1}{H}\:{\sum\:}_{i=1}^{H}{x}_{c}(i,w)\:(\mathrm{y}\mathrm{i}\mathrm{e}\mathrm{l}\mathrm{d}\mathrm{i}\mathrm{n}\mathrm{g}\:{Z}_{w}\: \in \:{R}^{1\times\:W\times\:C}$$

These are then concatenated in Eq. (6).

6 $$\:F=Concat({Z}_{h}\:,\:{Z}_{w})\: \in \:{R}^{(H+W)\times\:1\times\:C}$$

2. Shared 1D Convolutional Transformation: The concatenated features F are passed through a shared 1 × 1 convolutional layer $\:f\: \in \:{R}^{\left(H+W\right)\times\:1\times\:C/r}$ (where r is the reduction ratio, e.g.^32^), followed by a non-linear activation function and batch normalization, as shown in Eq. (7).

7 $$\:f=ReLU\left(BatchNorm\right(Conv{1D}_{1\times\:1}\left(F\right)\left)\right)$$

3. Split and 1D Convolutional Gates: $\:f$ is then split back into horizontal ($\:{f}_{h} \in \:{R}^{H\times\:1\times\:C/r}$) and vertical ($\:{f}_{w} \in \:{R}^{W\times\:1\times\:C/r}$) components. Separate 1 × 1 convolutional layers transform $\:{f}_{h}$​ and $\:{f}_{w}$​ into attention weights $\:{g}_{h}$​ and $\:{g}_{w}$​, as defined in Eq. (8) and Eq. (9).

8 $$\:{g}_{h}=\:\sigma\:\left(Conv1{D}_{1\times\:1}\left({f}_{h}\right)\right) \in \:{R}^{H\times\:1\times\:C}$$

9 $$\:{g}_{w}=\:\sigma\:\left(Conv1{D}_{1\times\:1}\left({f}_{w}\right)\right) \in \:{R}^{1\times\:W\times\:C\:}\:$$

where σ denotes the sigmoid activation function.

4. Attention-Weighted Output: The final output $\:{X}_{out}$​ is obtained by element-wise multiplication of the original input feature map X with the attention weights $\:{g}_{h}$​ (broadcasted across the width) and $\:{g}_{w}$ (broadcasted across height), as shown in Eq. (10).

10 $$\:{X}_{out}=\:\:X\:\otimes\:{g}_{h}\:\otimes\:{g}_{w}$$

This positional and channel-aware attention, particularly when applied at the bottleneck, enables the network to better localize targets such as the fetal cerebellum, even in abstract feature spaces, significantly improving computer vision performance with minimal computational cost^33^.

Decoder path (expansive path): The decoder aims to reconstruct a high-resolution segmentation map from compressed, attention-enhanced features through a series of up-sampling operations that progressively increase the spatial resolution of the feature maps. Skip connections play a critical role by directly concatenating the rich, SE-enhanced feature maps from the corresponding encoder stage with the up-sampled features in the decoder. This mechanism ensures the recovery of fine-grained spatial details lost during down-sampling, significantly enhancing the precision of the final segmentation boundaries.

As part of our novel SS_CASE_UNet architecture, we introduce an additional SE block within each up-sampling unit in the decoder, positioned after the concatenation and subsequent convolutional layers. This strategic placement provides a second layer of feature refinement in the expansive path, allowing the model to adaptively select and amplify the most informative features as it reconstructs the segmentation mask.

Each up-sampling block typically consists of an UpSampling2D layer, followed by concatenation with the SE-enhanced skip features, two Conv2D layers with ReLU activation, and a dropout layer. The final output block performs one more UpSampling2D operation to restore the original image resolution, followed by two Conv2D layers and a final dropout layer. The ultimate output is a $\:1\times\:1$ Conv2D layer with a sigmoid activation, producing a single-channel probability map that indicates the likelihood of each pixel belonging to the fetal cerebellum. The overall reduction in filter counts throughout the decoder and attention blocks is a deliberate design choice aimed at optimizing for a lower parameter count and improved generalization when training with limited medical data.

### Semi-supervised training strategy: A multi-stage approach

**Fig. 3:**
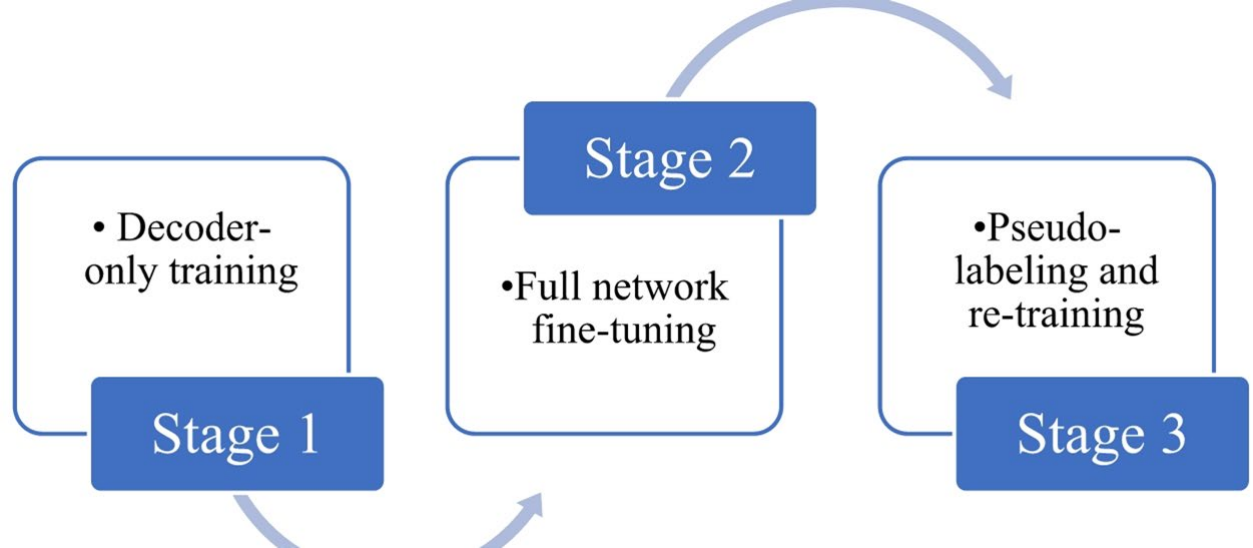
Illustration of the three training stages of the semi-supervised approach.

The core innovation of our proposed method lies in its multi-stage semi-supervised learning strategy^34^. This approach is explicitly designed to maximize the utility of our available data: a limited labeled dataset of 200 images alongside 200 unlabeled images. This strategy is particularly vital in medical imaging, where obtaining extensive expert annotations is notoriously costly and time-consuming^35^. Our entire strategy is organized into three distinct training stages, with dynamic data augmentation applied at each step to significantly enhance model learning and robustness. Figure 3 illustrates the complete three-stage training process.

Stage 1: Decoder-only training (transfer learning adaptation): In the initial phase, the weights of the pre-trained EfficientNetB0 encoder layers are frozen, meaning they remain fixed and are not updated during backpropagation. Only the randomly initialized decoder layers of the CASE_UNet, including the newly introduced attention blocks, are trained. This targeted training enables the model to quickly adapt its up-sampling and localization capabilities to the specific task of fetal cerebellum segmentation, leveraging the robust, general-purpose feature representations already learned by the pre-trained encoder. This stage provides an initialization for the decoder, aligning it with the encoder’s feature space while preventing drastic changes to the powerful pre-trained features. During this phase, a comprehensive data augmentation strategy (detailed in “Comprehensive augmentation (stages 1 & 2)”) is applied to the labeled training data.

Stage 2: Full network fine-tuning: After the initial decoder training, all layers of the CASE_UNet model, including the EfficientNetB0 backbone and all attention blocks, are unfrozen. The entire network undergoes a comprehensive fine-tuning phase with a significantly reduced learning rate. This global fine-tuning allows for minute adjustments throughout the architecture, enabling the pre-trained encoder to adapt to the subtle and unique features of fetal ultrasound images. This fosters an optimal collaboration between the encoder, decoder, and attention mechanisms, enhancing segmentation accuracy on the initial labeled training set of 150 images. The same comprehensive data augmentation strategy used in Stage 1 is applied here to further improve generalization.

**Fig. 4:**
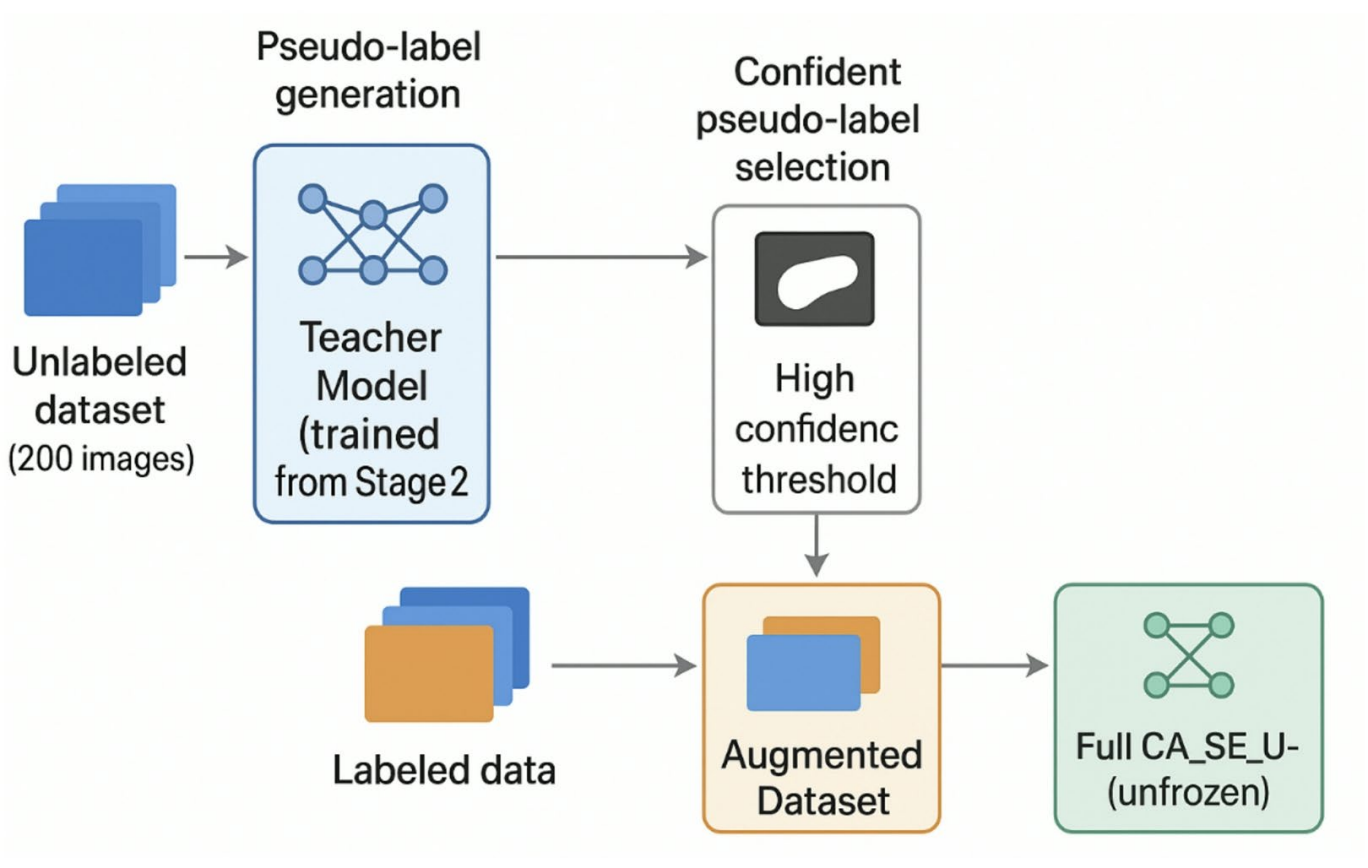
Illustration of pseudo-labeling and re-training stage.

 Stage 3: Pseudo-labeling and re-training (unlabeled data exploitation): This final stage is the cornerstone of our semi-supervised approach, explicitly designed to harness the valuable information latent within the unlabeled dataset^36^. This process is crucial for enhancing the model’s robustness and generalization capabilities beyond what is achievable through purely supervised learning. Figure 4 illustrates the complete three-step workflow of this stage.

- Pseudo-label generation: The well-tuned model from Stage 2 serves as our initial “teacher” model^37^. We first use this teacher model to perform inference on the entire pool of 200 unlabeled images. The resulting output probability maps provide the raw, initial predictions for the unannotated data.
- Confident pseudo-label selection: To ensure the quality and reliability of the generated pseudo-labels, a rigorous filtering process is applied. Only pseudo-labels that meet two strict criteria are selected: (i) High confidence threshold: We impose a high confidence threshold, accepting only pseudo-labels where the predicted probability for each foreground pixel (cerebellum) exceeds 0.95. This stringent criterion minimizes the propagation of noisy or incorrect labels. (ii) Minimum pixel count: Pseudo-masks with an extremely small number of predicted foreground pixels (fewer than 10 pixels) are discarded. This measure effectively filters out spurious or anatomically irrelevant predictions, ensuring that only meaningful structures are included.
- Dataset augmentation and re-training: The selected high-confidence pseudo-labeled images are combined with all available original labeled images. This process creates an expanded and diverse training dataset. The entire CASE_UNet model, with all layers unfrozen, is then re-trained using this augmented dataset. This re-training phase is critical as it exposes the model to a larger and more varied set of examples, preventing overfitting to the initial labeled dataset and fundamentally enhancing its ability to generalize to unseen data. This iterative process of generating and incorporating high-confidence pseudo-labels allows the model to learn effectively from unannotated data, directly addressing the challenges associated with limited annotated medical imaging datasets. For this specific stage, a simplified data augmentation strategy (detailed in “Simplified augmentation (stage 3 - pseudo-labeling)”) is applied to avoid amplifying potential pseudo-label noise and to ensure that the model focuses on fundamental features from the pseudo-labeled data without being distracted by complex transformations.

## Result analysis

This section outlines the experiments to validate the proposed method for fetal cerebellum segmentation. It begins with a comprehensive overview of the dataset utilized, detailing the characteristics and augmentation of the data. Subsequently, implementation settings and evaluation metrics are articulated to assess the efficiency and accuracy of the proposed methodology. The final subsection presents an analysis of the results obtained from the experiments.

### Dataset

We utilized the publicly available ultrasound image dataset, FETAL_PLANES_DB^38^, originally compiled by Burgos-Artizzu et al. This high-quality resource was collected from two different hospitals using various operators and ultrasound machines, resulting in significant diversity in image quality and acquisition protocols. The meta-information, including patient number, ultrasound machine, operator, and gestational age distribution, is publicly accessible alongside the dataset. The collection features six categories of images, with fetal brain images further categorized into trans-thalamic, trans-cerebellum, and trans-ventricular planes.

**Fig. 5:**
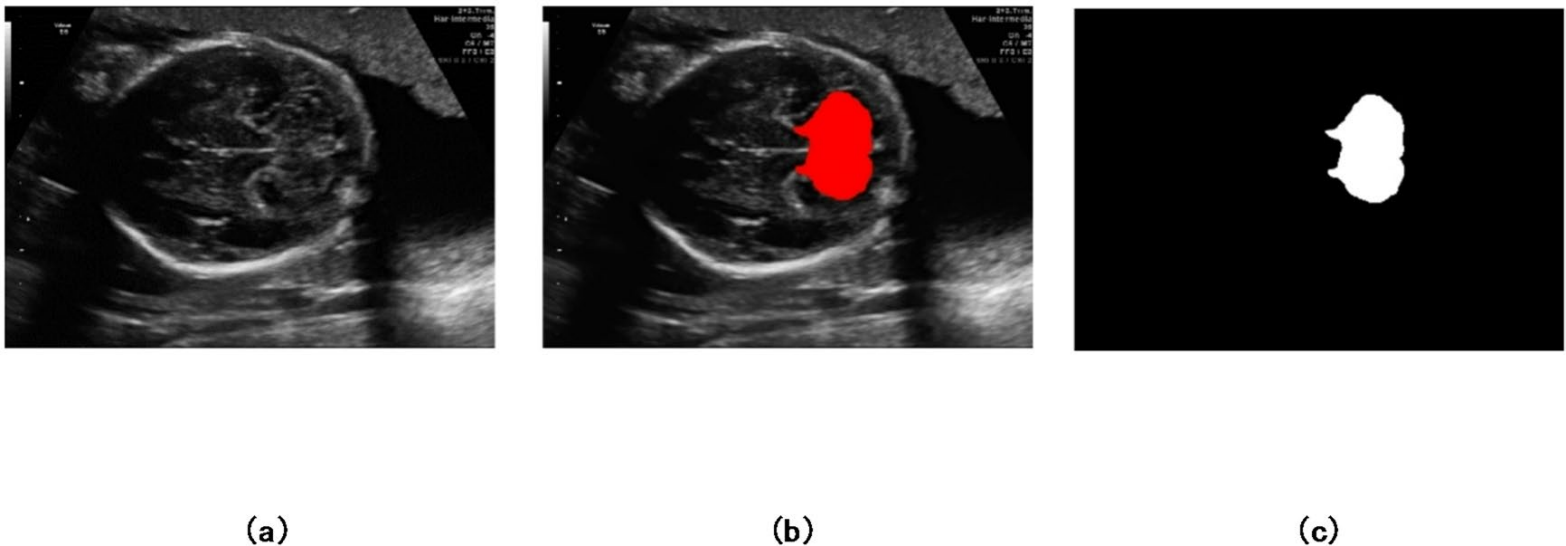
Illustration of the cerebellar area labeling process. (**a**) Original ultrasound image, (**b**) cerebellar area determined by the expert, and (**c**) corresponding ground truth mask.

Our study specifically used 400 images from the trans-cerebellum plane, where the cerebellar area was visible. An expert performed the original manual labeling for classification. For our segmentation task, an expert then manually annotated the cerebellar boundaries in 200 of these images to ensure maximal annotation consistency and clinical relevance. The remaining 200 images were retained to serve as the unlabeled data pool for our semi-supervised strategy. The progression of this specialized labeling process is illustrated in Fig. 5. The 200 fully labeled images were then split for supervised training and evaluation as follows: 75% (150 images) for training, 20% (40 images) for testing, and 5% (10 images) for validation.

Finally, we confirm that Institutional Review Board (IRB) approval was not required for this study because the analysis was performed on a publicly available, de-identified dataset. The dedicated cerebellar annotations used for the segmentation task were completed by the authors.

###  Data augmentation

To mitigate the challenges posed by limited annotated data and to enhance the model’s robustness and invariance to real-world variations, we employ a comprehensive and context-preserving data augmentation strategy^39^. Traditional augmentation methods were chosen for their safety, efficiency, and control, preserving diagnostic details without risking distortions. Unlike GANs, these basic transformations prevent artifacts and are effective with smaller datasets, maintaining data integrity, which is crucial for accurate clinical models^40^. This strategy is dynamically applied during training via a custom data generator, ensuring efficient memory usage and continuous data diversity. The augmentation pipeline is bifurcated into two distinct modes: a “Comprehensive Augmentation” strategy for the initial supervised training stages (Stage 1 and Stage 2) and a “Simplified Augmentation” strategy specifically tailored for the semi-supervised re-training phase (Stage 3).

#### Comprehensive augmentation (stages 1 & 2)

This strategy is designed to expose the model to a wide variety of realistic transformations, thereby enhancing its robustness and generalization capabilities when learning from high-quality labeled data. Each original training image-mask pair undergoes a structured sequence of transformations, culminating in 32 unique augmented pairs per original sample.

The pipeline consists of noise reduction, morphological operations, and geometric transformations. Here is a detailed explanation of each step:

Noise reduction: Each input image first undergoes a median-blur operation with a kernel size of five. This non-linear filter is highly effective at reducing speckle noise, a common artifact in ultrasound images, thereby improving image quality for subsequent feature extraction.

Morphological operations: To refine and diversify the structural representations of the cerebellum mask, various morphological operations are applied. These include dilation (expanding foreground regions), erosion (shrinking foreground regions), opening (erosion followed by dilation, useful for removing small objects or noise), and closing (dilation followed by erosion, useful for filling small holes or gaps). Each of these four operations is executed with both 3 × 3 and 5 × 5 kernels, generating eight distinct morphological variants for the mask. These variants are then paired with the noise-reduced image.

Geometric transformations: To enhance spatial robustness and simulate natural anatomical variations, the following geometric transformations are applied to each of the eight morphological variants (image-mask pairs): (i) Original scale: The image and mask are included without any additional resizing. (ii) Zoom (1.2x scale): Images and their corresponding masks are resized by a factor of 1.2 (120% scale) using bilinear interpolation for images and nearest-neighbor for masks. They are then centrally cropped back to the original 256 × 256 resolution. This simulates variations in anatomical scale and improves the model’s ability to handle different magnifications. (iii) Horizontal flip: Images and their masks are flipped along the vertical axis. (iv) Vertical flip: Images and their masks are flipped along the horizontal axis.

**Fig. 6:**
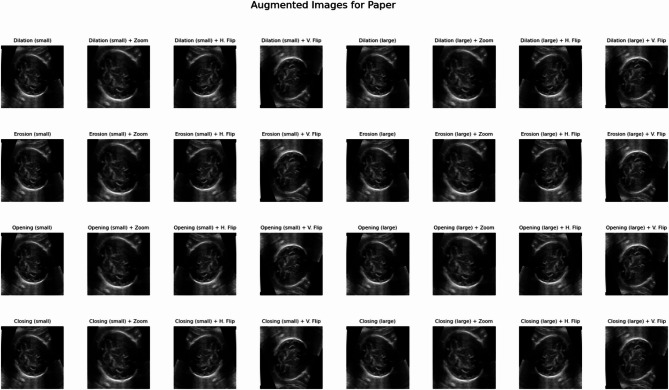
Examples of comprehensive data augmentation. Visualizations of augmented images generated from a single original input.

This combination of eight morphological variants and four geometric transformations yields a total of 8 × 4 = 32 unique augmented image-mask pairs from each original sample. Figure 6 showcases the generated augmented samples.

####  Simplified augmentation (stage 3-pseudo-labeling)

For the crucial pseudo-labeling re-training stage, a more controlled and simplified augmentation strategy is employed. This is a deliberate choice to prevent the amplification of potential noise or inaccuracies inherent in the generated pseudo-labels. This strategy produces eight augmented pairs for each original image and mask, comprising:

Noise reduction: A median blur (kernel size five) is applied to the image to effectively reduce noise, similar to the comprehensive strategy.

Morphological operations: Four core morphological operations—dilation, erosion, opening, and closing—are applied to the mask using a small (3 × 3) kernel. This approach mitigates significant shape changes compared to larger kernels or multiple kernel sizes, preserving the integrity of pseudo-labeled structures more reliably.

Geometric flips: Only horizontal and vertical flips are applied to both the noise-reduced image and its original mask. Zoom is intentionally excluded from this simplified set to minimize potential distortions in the pseudo-labels.

Minor intensity adjustment: An additional augmentation involves a slight brightness and contrast adjustment, such as random scaling of pixel values within a small range of 0.8 to 1.2. This subtly alters the image’s appearance without distorting its underlying structure, enhancing illumination robustness.

**Fig. 7:**
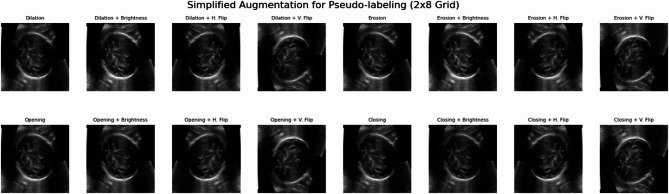
Examples of simplified data augmentation. Visualizations of augmented images generated from an image.

This reduced set of transformations allows the model to learn fundamental features from the pseudo-labeled data more reliably, without the distraction of overly complex or potentially noisy transformations during this sensitive re-training phase. Figure 7 showcases the generated augmented samples.

### Training

We applied transfer learning in our study by utilizing the network parameters of a pre-trained U-Net with an EfficientNetB0 backbone, which was initially trained on the ImageNet dataset. Our primary objective was to evaluate its effectiveness in predicting fetal cerebellar segmentation after training on our specific dataset. Our semi-supervised method employed a three-stage training process, incorporating dynamic data augmentation at each step to enhance model robustness. In Stage 1, we trained the unfrozen decoder layers for 20 epochs. This was followed by Stage 2, which involved a full network fine-tuning phase lasting 50 epochs. Finally, Stage 3 included pseudo-labeling and re-training for 30 epochs, a duration selected to balance adequate model convergence with the prevention of overfitting to our dataset. This comprehensive process aimed to fine-tune the model parameters for improved predictive accuracy in our task.

All models were developed utilizing TensorFlow v2.15, and the experiments were conducted on the Google Colab platform.

Loss functions for semi-supervised cerebellar segmentation: Our semi-supervised method utilizes a combined loss function to effectively train the model using both labeled and unlabeled data. This function balances a supervised Dice loss for annotated samples with an unsupervised Mean Squared Error (MSE) consistency regularization loss for unannotated samples.

Supervised segmentation loss: For the labeled data, we employ the Dice Loss, a standard metric widely recognized in medical image segmentation for effectively addressing class imbalance. The Dice Loss quantifies the overlap between the prediction and the ground truth, as shown in Eq. (11).

11 $$\:{\mathcal{L}}_{Dice}\:\left(P,G\right)=\frac{2\sum\:_{i}{P}_{i}{G}_{i}}{\sum\:_{i}{P}_{i}^{2}+\sum\:_{i}{G}_{i}^{2}}$$

where $\:{P}_{i}\:$is the predicted probability and $\:{G}_{i}$ is the ground truth label for pixel$\:\:i$.

Unsupervised consistency regularization loss (mean squared error): To leverage unlabeled data, we use a consistency regularization loss. This enforces stable predictions for unlabeled inputs under perturbations, calculated as the MSE between two passes of the same input in Eq. (12).

12 $$\:{\mathcal{L}}_{MSE}\:\left({P}_{1}\:,\:{P}_{2}\right)=\frac{1}{N}\sum\:_{i}{({P}_{1,i}-\:{P}_{2,i})}^{2}$$

where $\:{P}_{1,i}$ and $\:\:{P}_{2,i}\:$ are softmax outputs from two passes of pixel $\:i$, and $\:\boldsymbol{N}$ is the total number of pixels.

Total combined loss: The overall training loss *L* is a weighted sum, balancing supervised accuracy and unsupervised consistency, as shown in Eq. (13).

13 $$\:L\:=\:\:{\mathcal{L}}_{\mathrm{D}\mathrm{i}\mathrm{c}\mathrm{e}}+\:{\uplambda\:}{\mathcal{L}}_{\mathrm{M}\mathrm{S}\mathrm{E}}$$

here, λ is a hyperparameter controlling the weight of the consistency term, often annealed during training. This combined approach optimizes for robust and generalizable cerebellar segmentation.

### Model evaluation metrics

To rigorously evaluate the performance of our SS_CASE_UNet model for fetal cerebellar segmentation, we employ Accuracy, Precision, Recall, Jaccard Similarity (JS), and Dice Similarity Coefficient (DSC) as five metrics. These quantitative metrics assess various aspects of segmentation accuracy, providing a comprehensive understanding of the model’s capabilities.

DSC, also known as the F1-score or Sørensen-Dice index, is a widely used metric in medical image segmentation. It measures the spatial overlap between the predicted segmentation (P) and the ground truth (G), ranging from 0 (no overlap) to 1 (perfect overlap). Given its direct relation to the Dice Loss used during training, it serves as a primary indicator of segmentation quality. The formulas for Accuracy, Precision, Recall, JS, and DSC are defined in Eq. (14) to Eq. (18).

14 $$\:Accuracy=\:\frac{TP\:+\:TN\:}{TP\:+\:FP\:+\:TN\:+\:FN}$$

15 $$\:Precision=\:\frac{TP\:}{TP\:+\:FP\:}$$

16 $$\:Recall=\:\frac{TP\:}{TP\:+\:FN}$$

17 $$\:Jaccard\:Similarity=\:\frac{TP\:\:}{TP\:+\:FP\:\:+\:FN}$$

18 $$\:DSC\:\:\left(P\:,\:G\right)=\frac{2\:|P\cap\:G|\:}{|P|+\:|G|}=\frac{2\:\times\:TP}{2\:\times\:TP+FP+FN}$$

where TP, TN, FP, and FN represent the number of True Positives, True Negatives, False Positives, and False Negatives, respectively.

### Segmentation results

Our work introduces SS_CASE_UNet, a novel semi-supervised deep learning architecture designed for accurate fetal cerebellar segmentation. Its superior performance builds on the U-Net framework by integrating Squeeze-and-Excitation (SE) and Coordinate Attention (CA) blocks. This combined attention mechanism, along with our semi-supervised training strategy that leverages both labeled and unlabeled data, significantly enhances segmentation accuracy in challenging medical image analysis.

To demonstrate the effectiveness of SS_CASE_UNet, we conducted a rigorous comparative analysis against several state-of-the-art algorithms. These included FCRBU-Net^23^, an enhanced U-Net utilizing fully connected residual blocks; ECAU-Net^18^, which integrates Efficient Channel Attention for improved efficiency; and Dual_CNN^11^, a two-stage approach that focuses first on plane localization before segmentation. We ensured a fair comparison by successfully reproducing these algorithms and conducting all experiments with identical training strategies and parameter settings. Table 2 provides a quantitative summary of the overall cerebellar segmentation results, highlighting the performance of each method.

As demonstrated in Table 2, our proposed SS_CASE_UNet model consistently outperforms all other state-of-the-art methods on the test dataset, achieving the highest scores across all five evaluation criteria (Accuracy, Precision, Recall, Jaccard Similarity (JS), and Dice Similarity Coefficient (DSC)).

Specifically, SS_CASE_UNet achieved an impressive Accuracy of 99.08% and a strong DSC of 87.65%. The model’s high accuracy is particularly noteworthy given the significant class imbalance in the dataset. Compared to FCRBU-Net^23^, the next best-performing model, SS_CASE_UNet demonstrated significant gains: an increase in Accuracy by 0.42, JS by 5.22, and DSC by 1.19. These robust gains underscore the effectiveness of our approach in complex fetal cerebellum segmentation. This superior performance is a direct result of the deliberate integration of our attention mechanisms, a justified increase in complexity even compared to the most lightweight competitor, Dual_CNN^11^.

**Table 2:** Comparisons of different models on the test dataset.

Method	Metrics					#Params	Average Inference Time (ms)
	Accuracy	Pre	Rec	JS	DSC		
U-Net	97.80	84.26	74.18	65.08	78.89	10 M	1972.36
ECAU-Net ^18^	98.03	90.85	79.91	76.17	85.04	31 M	2734.51
FCRBU-Net^23^	98.66	92.61	79.95	76.54	85.74	33 M	5465.72
Dual_CNN^11^	97.55	83.89	75.45	67.57	79.34	**3M**	1017.52
SS_CASE_UNet	**99.08**	**93.49**	**82.34**	**81.76**	**87.65**	6.7 M	489.57

The best values are in bold.

**Fig. 8:**
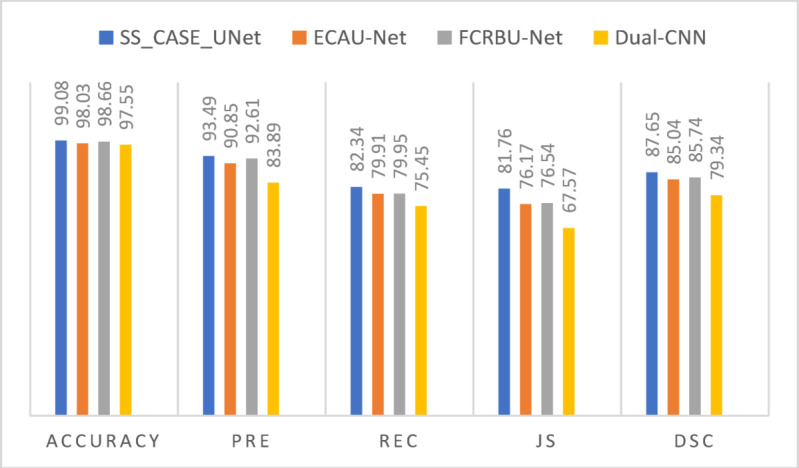
Quantitative Performance Comparison of SS_CASE_UNet and State-of-the-Art Deep Learning Models.

This performance advantage is achieved through the combination of Squeeze-and-Excitation (SE) blocks for channel recalibration and the Coordinate Attention (CA) block for precise spatial awareness. This enables our model to extract more discriminative and contextually rich features from noisy ultrasound images—a capability that simpler, lower-parameter models often lack. This strategic architectural design is the primary reason SS_CASE_UNet achieves better results without the excessive parameter increase seen in other advanced U-Net variants. Building on the quantitative summary in Table 2; Fig. 8 visually represents the segmentation results, offering a clear comparative analysis of SS_CASE_UNet’s performance against other established deep learning models across all evaluation metrics.

**Fig. 9:**
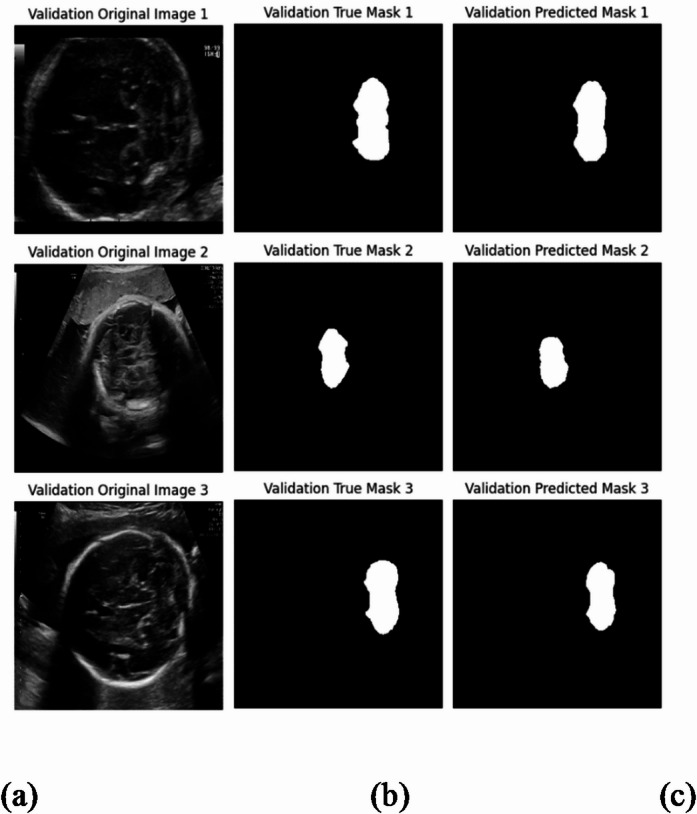
The performance of SS_CASE_UNet with test-time augmentation. (**a**) The original ultrasound image, (**b**) the ground-truth, (**c**) the segmentation results of SS_CASE_UNet.

To enhance prediction consistency and robustness—a critical requirement for clinical medical imaging tasks—we implemented Test-Time Augmentation (TTA). This technique applies fundamental geometric operations (such as horizontal and vertical flips) to each test image. With TTA applied, SS_CASE_UNet achieved a DSC of 85.74% (along with an Accuracy of 98.26%, Precision of 92.61%, Recall of 80.82%, and JS of 80.03%). As Fig. 9 visually confirms, our model consistently and accurately recognizes the fetal cerebellar area, highlighting its enhanced robustness and generalization despite subtle variations in image orientation and presentation.

The number of model parameters and the resulting inference time are critical factors for practical clinical deployment. FCRBU-Net, with the highest parameter count of 33 million, is consequently the slowest model, requiring 5465.72 ms for a single prediction—a speed that severely limits its real-time application. In stark contrast, our SS_CASE_UNet, with only 6.7 million parameters, achieves optimal speed, boasting the fastest inference time of just 489.57 ms. Although Dual_CNN has the lowest parameter count at 3 million, its prediction time is significantly slower at 1017.52 ms. This disparity arises from Dual_CNN’s computationally expensive dual-network design, underscoring the superior real-time efficiency of our streamlined, single-pipeline SS_CASE_UNet architecture.

This optimal balance of performance and efficiency is achieved through a deliberate architectural strategy: while lightweight SE and CA attention blocks were integrated, the overall parameter count remains lower than that of a standard U-Net baseline primarily due to a strategic reduction in the number of filters within the decoder path (e.g., reducing filter counts from 128 to 8 in the final stage). This careful parameter optimization ensures superior segmentation performance with a practical and optimized model size.

**Fig. 10:**
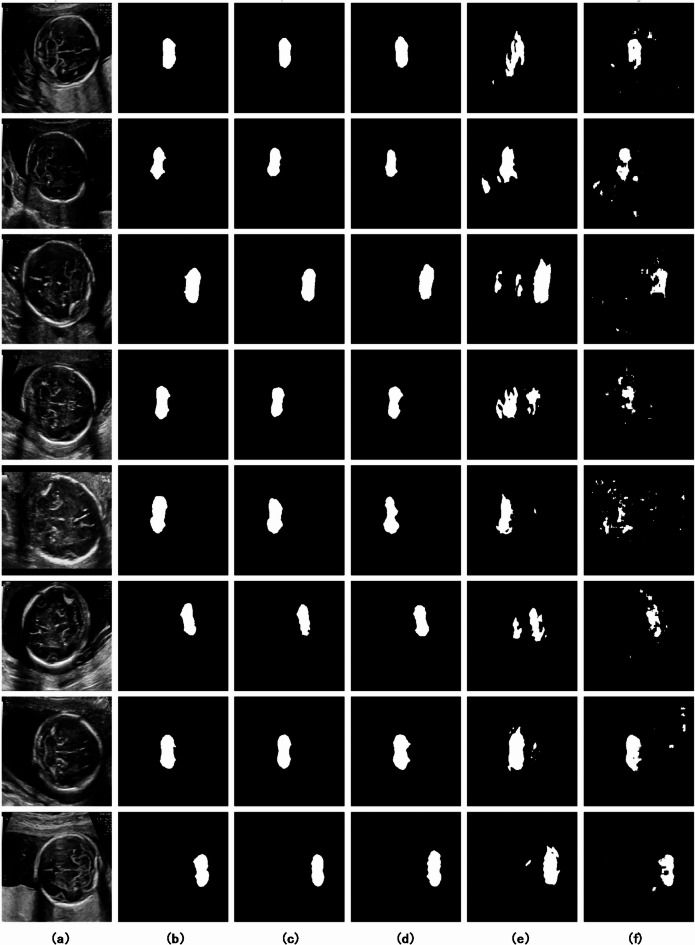
Comparison of fetal head segmentation by different deep learning models. (**a**) The original ultrasound image, (**b**) the ground-truth, and the segmentation results of (**c**) SS_CASE_UNet, (**d**) FCRBU-Net, (**e**) ECAU-Net, and (**f**) Dual_CNN.

Figure 10 offers a crucial visual comparison of fetal cerebellum segmentation across all models, displaying the original ultrasound images, corresponding ground truth, and binary segmentation results. SS_CASE_UNet demonstrates superior segmentation results, consistently resembling the ground truth, which attests to its high fidelity. A notable limitation of the comparative methods is their difficulty in handling inherent ultrasound noise, often resulting in fragmented predictions, false positives, and a lack of boundary continuity. For example, in Row 2, while models like ECAU-Net and Dual_CNN present fragmented or inaccurate predictions, SS_CASE_UNet effectively delineates the complex cerebellar boundary with high fidelity. Furthermore, Row 5 strongly illustrates SS_CASE_UNet’s robustness in segmenting the cerebellum, even under challenging image quality, producing a complete and accurate mask where other methods exhibit significant omissions or false positives. This ability to maintain the structural integrity and continuity of the segmented cerebellum highlights the model’s resistance to common failure modes seen in less robust approaches.

To validate our specialized semi-supervised strategy, we compared SS_CASE_UNet with SEMI-PLC^29^, a well-known and general SSL framework. While SEMI-PLC uses a dual-subnetwork architecture and stability masks to generate consensus pseudo-labels, our approach relies on a single, improved attention network coupled with a fully filtered single-step pseudo-labeling mechanism. As shown in Table 3, the SEMI-PLC method achieved a final DSC of only 72.32%. This poor performance is likely due to its general architecture, which struggles with the high speckle noise and low contrast inherent in fetal ultrasound images. In contrast, our SS_CASE_UNet achieved a DSC of 87.65%, representing a significant improvement of 15.33%. This significant gap provides strong evidence that the integration of SE and CA attention blocks is crucial for extracting reliable features from noisy ultrasound data.

**Table 3:** Performance metrics of the comparative Semi-Supervised segmentation framework (SEMI-PLC^29^.

Methods		Metric				
		Accuracy	Precision	Recall	JS	DSC
SEMI-PLC^29^	Subnet A	98.19	83.56	62.72	55.48	71.03
	Subnet B	98.26	82.04	67.61	58.63	73.61
	Average Result	98.23	82.80	65.17	57.06	72.32
SS_CASE_UNet		**99.08**	**93.49**	**82.34**	**81.76**	**87.65**

The best values are in bold.

**Fig. 11:**
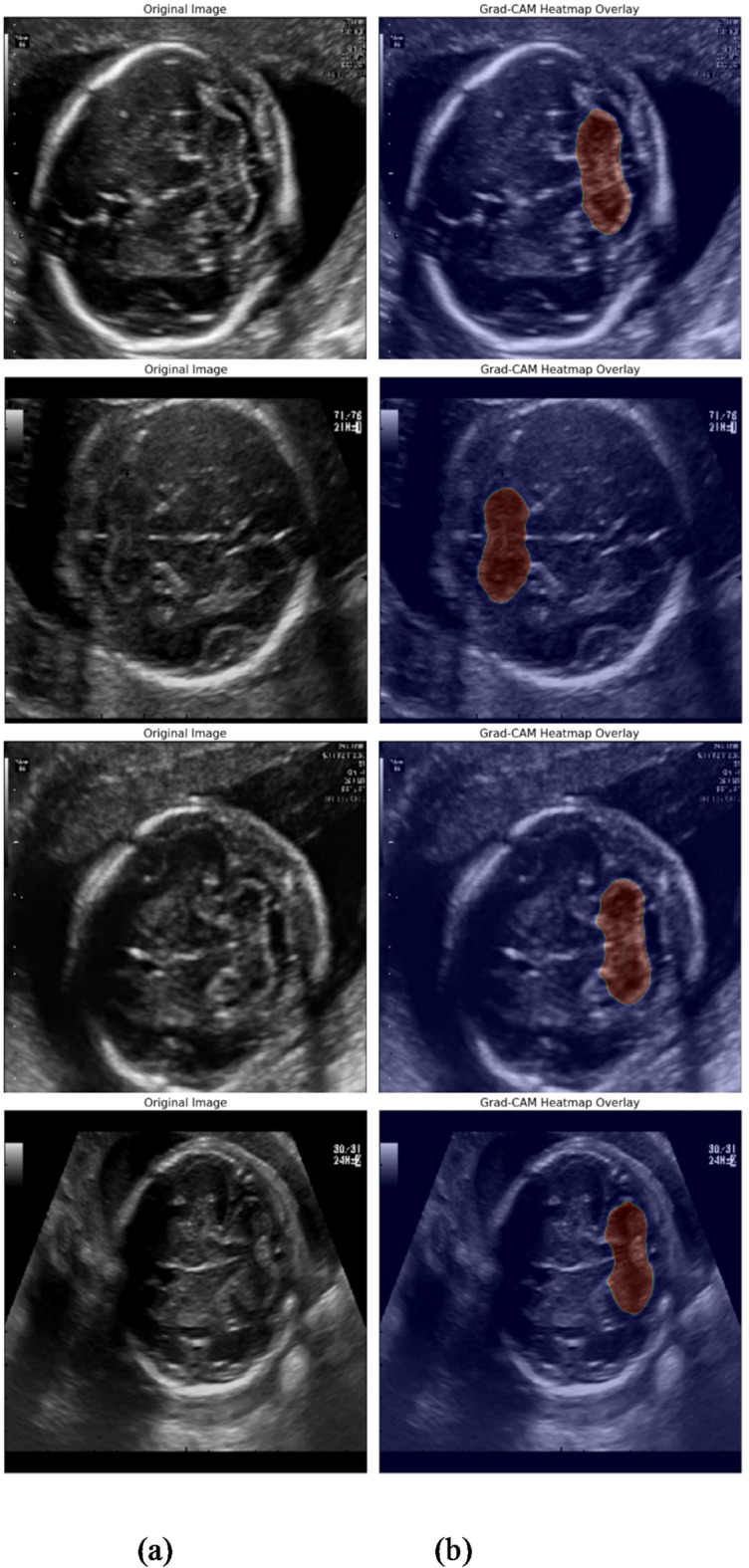
Visual representation of the SS_CASE_UNet model’s performance using heatmaps. (a) Original ultrasound image, and (b) Grad-CAM heatmap.

To further elucidate the internal workings and interpretability of our SS_CASE_UNet model, we utilize Gradient-weighted Class Activation Mapping (Grad-CAM)^41^ heatmap to visualize the regions of an image that most strongly influence the model’s segmentation decisions. Figure 11 presents representative heatmaps overlaid on the original ultrasound images, showcasing the spatial focus of our model during inference. These visualizations demonstrate how SS_CASE_UNet’s integrated attention mechanisms (SE and CA blocks) enable it to concentrate precisely on the fetal cerebellum, even amidst surrounding noise and complex anatomical structures. The heatmaps confirm that the model effectively identifies and prioritizes the relevant pixels, providing a deeper understanding of its robust segmentation performance. This visual evidence supports the efficacy of our architectural design in enhancing discriminative feature learning.

###  Ablation experiments

In addition to verifying the effectiveness and generalization capabilities of our proposed method through comparative experiments (as discussed in “Segmentation results”), we conducted an ablation study to rigorously evaluate the contribution and necessity of each component within the SS_CASE_UNet framework. The quantitative results of these experiments are summarized in Tables 4 and 5.

Our analysis began by establishing the baseline performance of the standard U-Net model, which achieved a Dice Similarity Coefficient (DSC) of 78.89%.

Upon integrating the Coordinate Attention (CA) block into the U-Net, we formed the CA-UNet model, which demonstrated substantial improvements across all evaluated metrics. As summarized in Table 4, the CA-UNet yielded a DSC of 86.98% (an 8.09% improvement) compared to the baseline, clearly demonstrating the CA block’s effectiveness in directing the network’s attention to critical spatial features. Similarly, the inclusion of the Squeeze-and-Excitation (SE) block, resulting in the SE-UNet model, led to significant performance gains, achieving a DSC of 85.20% (a 6.31% improvement over the baseline). This marked improvement highlights the SE block’s ability to recalibrate channel-wise feature responses, optimizing information flow within the network.

We then experimented with a fully supervised CASE-UNet model, which incorporates both the CA and SE blocks and is trained exclusively on the limited labeled dataset. The results confirm the synergistic collaboration between the two attention mechanisms, with the model achieving a DSC of 87.16%, surpassing the performance of either individual block configuration.

**Table 4:** Results of the ablation experiments on the test set.

Model	Blocks		Metrics					#Params
	CA_Block	SE_Block	Accuracy	Pre	Rec	JS	DSC	
U-Net	--	--	97.80	84.26	74.18	65.08	78.89	9.67 M
CA-UNet	✔	--	98.91	92.84	81.93	76.95	86.98	9.83 M
SE-UNet	--	✔	98.65	91.05	80.11	74.24	85.20	6.57 M
CASE-UNet	✔	✔	98.97	92.94	82.06	77.27	87.16	6.74 M
SS_CASE_Unet	✔	✔	99.08	93.49	82.34	81.78	87.65	6.74 M

To quantify the specific advantage of our multi-stage semi-supervised strategy, we conducted an ablation study focused solely on the training process, as detailed in Table 5. We used the supervised CASE-UNet (DSC 87.16%) as our baseline, representing the model’s performance with labeled data only.

When processing all 200 unlabeled images with pseudo-labels but without rigorous confidence and size filtering (Naive_CASE-UNet), the performance showed only a modest increase (DSC of 87.24%). This highlights the significant risk of error propagation from noisy ultrasound data. By applying our proposed strict filtering criteria (0.95 probability and a minimum size of 10 pixels), the full SS_CASE_UNet model achieved the highest performance metrics, with a final DSC of 87.65%. This represents an overall improvement of + 0.49 points over the supervised baseline, clearly demonstrating that the sequential, high-fidelity pseudo-labeling in Stage 3 is responsible for the majority of the final performance gains.

This analysis confirms that the superior performance of SS_CASE_UNet results from the synergistic combination of both the optimized attention-enhanced architecture and the carefully designed, filtered semi-supervised learning process.

**Table 5:** Ablation study of the Multi-Stage Semi-Supervised training Pipeline.

Model	Metrics				
	Accuracy	Pre	Rec	JS	DSC
CASE-UNet	98.97	92.94	82.06	77.27	87.16
Naive_CASE-UNet	98.99	93.06	82.11	77.36	87.24
SS_CASE_UNet	99.08	93.49	82.34	81.78	87.65

## Discussions

The accurate and efficient segmentation of the fetal cerebellum from ultrasound images is crucial for prenatal diagnostics, enabling early detection of neurological abnormalities and precise biometric measurements. However, as highlighted in the introduction, this task is inherently challenging due to speckle noise, varying image quality, complex anatomical structures, and significant logistical hurdles in acquiring large, expertly annotated datasets. Our proposed SS_CASE_UNet framework directly addresses these limitations by introducing a novel attention-enhanced U-Net architecture (CASE_UNet), coupled with a robust multi-stage semi-supervised training strategy.

Our comprehensive comparative analysis, quantitatively summarized in Table 2, clearly demonstrates that SS_CASE_UNet outperforms several established state-of-the-art models in fetal cerebellum segmentation. Achieving a DSC of 87.65%, along with high Accuracy (99.08%), Precision (93.49%), Recall (82.34%), and Jaccard Similarity (JS) (81.78%), our model establishes a new benchmark for this challenging task. The notable 2.23% point improvement in DSC over FCRBU-Net^23^, a leading baseline, underscores the effectiveness of our integrated approach in capturing the intricate boundaries and challenging features of the fetal cerebellum. Visually, as depicted in Fig. 10, SS_CASE_UNet consistently produces segmentation masks that closely resemble the ground truth, exhibiting superior boundary delineation and reduced artifacts compared to ECAU-Net^18^ or Dual_CNN^11^, especially in cases with low contrast or partial occlusions.

The rigorous ablation study, detailed in Table 4, provides critical insight into the individual contributions and synergistic effects of the attention blocks. Both the Coordinate Attention (CA) block and the Squeeze-and-Excitation (SE) block) individually enhance the baseline U-Net’s performance, validating their complementary roles in refining feature representations. The CA block’s ability to embed precise positional information is crucial for accurately localizing the cerebellum, while the SE block’s dynamic channel-wise feature recalibration is vital for emphasizing salient features and suppressing the influence of noise inherent in the ultrasound environment. The combined deployment of both blocks in the supervised CASE_UNet results in further incremental improvement in DSC, confirming their indispensable cooperation.

Furthermore, a key architectural advantage of SS_CASE_UNet is its optimized balance between model complexity and clinical efficiency. While models like FCRBU-Net^23^ have significantly higher parameter counts (e.g., 33 M), potentially limiting real-world deployability, our SS_CASE_UNet maintains a competitive 6.7 million parameters and boasts the fastest inference time of 489.57 ms. Although Dual_CNN^11^ has fewer parameters, its dual-network design makes its prediction time more than double that of our streamlined, single-pipeline architecture. This efficiency is achieved through the strategic use of parameter-efficient SE and CA blocks, combined with a deliberate reduction of filters in the decoder path, enabling our model to generalize effectively without the computational burden of larger models.

A core innovation of our work is the multi-stage semi-supervised training strategy. This strategy employs a Teacher-Student model for pseudo-label generation, sharing a conceptual link with methods like the ASC framework^28^. However, SS_CASE_UNet is specifically designed for ultrasound segmentation, addressing the challenge of noisy images through a refined Attention-Enhanced U-Net (CASE-UNet) architecture and a multi-stage training pipeline. This pipeline, which includes pre-training, fine-tuning, and re-training with strictly filtered, high-confidence pseudo-labels, is optimized for extracting reliable segmentation from noisy unlabeled ultrasound data. This represents a crucial distinction from the consistency-regularization often used in MRI-based UDA methods, such as ASC.

To validate SS_CASE_UNet, we compared it against the generalized SSL framework SEMI-PLC^29^. As detailed in Table 5, SEMI-PLC achieved a final DSC of only 72.32%, while SS_CASE_UNet attained an improvement of 15.33% points.

The implementation of Test-Time Augmentation (TTA) confirms the inherent stability and strong generalization capabilities of the model. TTA enhances the consistency and reliability of our segmentation, with Fig. 9 visually reinforcing the model’s ability to maintain precise segmentation despite subtle variations in image orientation.

Moreover, the interpretability offered by Grad-CAM heatmaps, visually represented in Fig. 11, provides crucial insights into our model’s decision-making process. These heatmaps clearly illustrate how SS_CASE_UNet precisely targets the cerebellar region, validating the effectiveness of the integrated attention mechanisms in guiding the network’s focus to relevant anatomical features, thereby minimizing the influence of noise and ambiguous boundaries.

In conclusion, SS_CASE_UNet represents a robust, highly accurate, and efficient solution for the automated segmentation of the fetal cerebellum. Its ability to achieve high performance, coupled with an optimized parameter profile and effective utilization of unlabeled data, positions it as a promising tool to augment prenatal screening processes. While our current focus has been on segmentation accuracy, the derived precise cerebellar masks lay a strong foundation for future work, including the automatic classification of cerebellar abnormalities, which could significantly aid clinicians in early diagnosis and intervention.

## Conclusion

This study introduces SS_CASE_UNet, an attention-enhanced, semi-supervised framework for accurate fetal cerebellum segmentation in ultrasound images. By integrating Squeeze-and-Excitation (SE) and Coordinate Attention (CA) blocks into a U-Net, our model effectively addresses challenges such as image noise, complex anatomical structures, and the scarcity of annotated data. Our results demonstrate that SS_CASE_UNet surpasses existing methods, achieving a Dice Similarity Coefficient (DSC) of 87.65%. Ablation studies confirm that both the CA and SE blocks are crucial for enhancing segmentation accuracy and robustness, showing a synergistic effect when used together. The model achieves this superior performance with a balanced and optimized parameter count, which is lower than the standard U-Net baseline, making it more practical for clinical deployment. The multi-stage semi-supervised training strategy and Test-Time Augmentation (TTA) were also key to our success, allowing the model to learn from limited labeled data and improve prediction consistency effectively. In conclusion, SS_CASE_UNet is a robust and efficient automated tool for fetal cerebellum assessment that addresses the key limitations of manual segmentation and data scarcity. For future work, the precise cerebellar segmentation masks generated by SS_CASE_UNet lay a strong foundation for advancing prenatal diagnostics. Our next critical step is to leverage this accurate anatomical localization to detect cerebellar abnormalities, with a focus on the automated identification of Dandy-Walker Malformation (DWM).

## Data Availability

The dataset analysed during the current study is available at https://zenodo.org/records/3904280.The generated code is available at [https://github.com/amenevatanparast/SS_CASE_UNet](https:/github.com/amenevatanparast/SS_CASE_UNet) .
